# Psychological factors mediating health-related quality of life in COPD

**Published:** 2014-03-25

**Authors:** O Popa-Velea, VL Purcarea

**Affiliations:** * Department of Medical Psychology, “Carol Davila" University of Medicine and Pharmacy, Bucharest; ** Department of Healthcare Marketing, Technology and Medical Devices, Medical Informatics and Biostatistics, “Carol Davila" University of Medicine and Pharmacy, Bucharest

**Keywords:** self-efficacy, optimism, COPD, health-related quality of life

## Abstract

COPD is a chronic disease that has not only a high prevalence and social costs, but is tightly connected to a significant decrease of health-related quality of life (HRQoL). The aim of this study was to evaluate the comparative impact on HRQoL of two psychological factors (self-efficacy, optimism) vs. classical medical determinants (forced expiratory volume in 1 second (FEV1), peak expiratory flow (PEF), functional impairment). 26 women and 28 men, aged 45-64 years old (mean = 58.1; standard deviation = 9.7), diagnosed with COPD and with self-reported dyspnea requiring medication were administered COPD Self-Efficacy Scale, LOT-R (Life Orientation Test - Revised) to evaluate optimism, Quality of Well-Being (QWB) Scale, as an accepted measure of HRQoL and Functional Impairment Scale (FIS), used to assess the deterioration of functionality in respiratory diseases. Their respiratory parameters (FEV1, PEF) were also measured, via spirometry. Results showed that self-efficacy and optimism were positively correlated to HRQoL (r = .34 (p < .05) and r = .29 (p < .05), respectively). A reduced model that eliminated the direct influence of respiratory parameters on HRQoL proved to be equally satisfactory in terms of predictor value, compared to the full model (that contained all studied variables) (χ2 = 0.067, ns). The functional impairment (FI) scores were inversely correlated with HRQoL (r = -.46, p < .01). These results have implications in considering self-efficacy and optimism as important factors when aiming HRQoL improvement in COPD, and for the inclusion of psychological interventions in the treatment plan of COPD patients.

Abbreviations COPD = chronic obstructive pulmonary disease; WHO = World Health Organization; HRQoL = health-related quality of life; PEF = peak expiratory flow; FEV1 = forced expiratory flow in one second; LOT-R = Life Orientation Test – Revised; QWB = Quality of Well-Being; FI = functional impairment; SE = self-efficacy; Opt. = optimism

## Introduction

Chronic obstructive pulmonary disease (COPD) is a lung disease characterized, pathologically, by the combination of chronic bronchitis and emphysema, and clinically by the chronic obstruction of lung airflow that interferes with normal breathing and is not fully reversible. The main symptoms include dyspnea, possibly accompanied by wheezing, and a persistent cough with sputum production. 

 COPD is an under-diagnosed lung disease, “the only major non-infectious disease that is increasing in prevalence and mortality nowadays" [**1**]. According to the World Health Organization (WHO) [**2**], 80 million people worldwide suffer from moderate to severe COPD, accounting for 100,000 deaths and 550,000 hospitalizations per year. The WHO predicts that by 2030, it will be the fourth largest cause of mortality in the world. In Romania, there are currently 600 – 800,000 cases at a population of 22 millions (2.30% ♀, 4.64% ♂) [**3**].

 COPD is one of the several chronic diseases for which health-related quality of life (HRQoL) – including both physical and psychosocial aspects - is considered an important treatment outcome [**4**]. According to the biomedical paradigm, the most powerful determinant of HRQoL should be the pulmonary function itself [**5**], in the sense that forced expiratory flow in one second (FEV1), PEF (peak expiratory flow), or the ratio PEF / vital capacity are generally proportional to HRQoL. However, some previous research [6,7] showed that these correlations are not always high or stable across the disease (with rs ranging from .01 to .32, most coefficients being below .20). Such results might suggest that the relationship of HRQoL to biomedical variables could be mediated by other factors, including psychological factors. In this sense, certain social cognitive models of Medical Psychology, which include cognitive variables as mediators for the effect of biomedical variables on quality of life, may have a real predictive value for the HRQoL in COPD [**8**]. For example, Kaplan et al. [**9**] suggest that there may be a strong correlation between self-efficacy and well-being scores in COPD patients. Scherer and Schmieder found, similarly, a relationship between self-efficacy and perceived pulmonary function in COPD [**10**].

### Aim, hypothesis

The purpose of this study was to determine the possible contribution of self-efficacy and optimism in mediating the relationship between the biomedical variables of pulmonary function and HRQoL. Our hypothesis was that both self-efficacy and optimism can account for a stronger correlation with HRQoL in COPD than the primary effect of the disease itself, expressed as the volume of air forcefully expired in 1s (FEV1).

## Material and method

The participants were 26 women and 28 men, aged 45-64 years old (mean = 58.1; standard deviation = 9.7), diagnosed with COPD (= chronic bronchitis and emphysema, with FEV1 < 80% of predicted, FEV1 / forced vital capacity < 70% of predicted) – mean FEV1 = 46,13% and with self-reported dyspnea requiring medication.

 The design of the study was transversal. Each participant was administered the following instruments: COPD Self-Efficacy Scale (a specific instrument to assess self-efficacy in COPD) [**11**], LOT-R (Life Orientation Test - Revised) - to evaluate optimism [**12**], Quality of Well-Being (QWB) Scale (as an accepted measure of HRQoL) [13,14], and Functional Impairment Scale (FIS) (largely used for measuring deterioration of functionality in respiratory diseases) [**15**]. FEV1, as a measure of assessing the objective deterioration of pulmonary function, was also measured via spirometry. The protocol of the study was designed according to the ethical demands of the research on human subjects, and all subjects gave their informed consent. The procedures followed were in accordance with the institutional guidelines of host institutions.

 Data were collected in two university hospitals of Bucharest and were introduced into a database using the SPSS 16.0 software. Correlations were performed between FEV1 and the scores of psychological variables, and a path analysis was designed to evaluate the degree to which a model including psychological variables could be a good predictor for the HRQoL at COPD patients. This model was tested versus a classical (“full") model, which included direct effects of FEV1 on HRQoL as well. Adequacy of fit between the two models was tested using chi-square statistic.

## Results

**Fig. 1:**
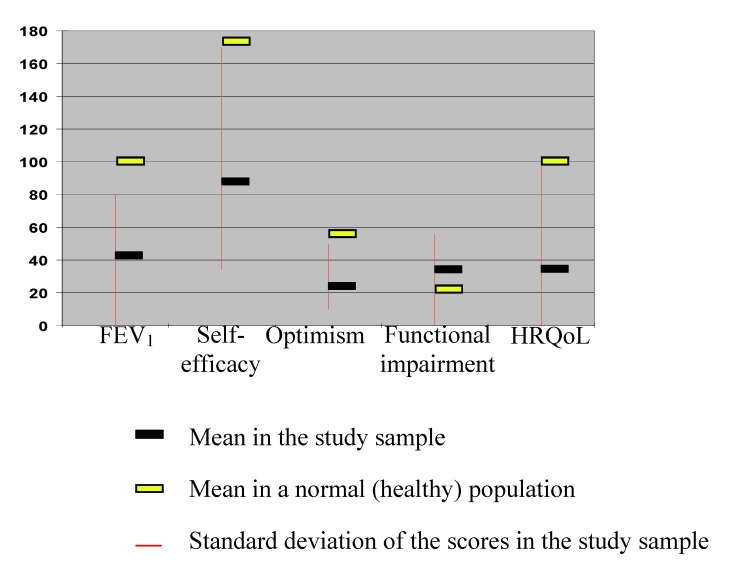
The distribution of studied variables (mean, SD) across the study sample, compared to the scores in a normal population

Descriptive data The synthesis of the mean scores of the psychosocial variables and HRQoL in the study sample, compared to the scores in a normal (healthy) population, is presented in Fig. 1.

 A deterioration of all scores, both for the independent variables and for the dependent variable (HRQoL), is conspicuous, this representing a further argument for the strong negative medical and psychological impact of COPD on daily functioning. 

 Statistical data 

 All direct Pearson correlations between study variables are figured in Table 1. 

**Table 1:** Direct Pearson’s correlations between study variables

Measure	FEV1	Self-efficacy	Optimism	FIS	HRQoL
FEV1	-	-.10	-.12	-.11	.18*
Self-efficacy		-	.22	-.37*	.34*
Optimism			-	- .23	.29*
FIS				-	-.46**
HRQoL					-
*p < .05 , **p < .01					

**Fig. 2:**
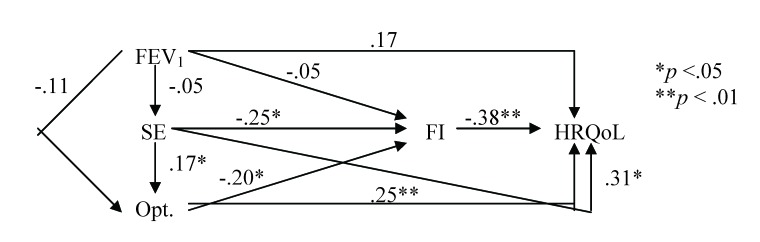
The “full" model (all-possible correlations between variables are figured). FEV1 = forced expiratory volume in 1s; FI = functional impairment; HRQoL = health-related quality of life; SE = self-efficacy; Opt. = optimism.

 One can notice the obvious positive correlations between the scores of psychological variables and the HRQoL. However, they are insufficient to disentagle the separate contribution of medical and psychosocial variables on HRQoL. In this scope, path analysis was used instead. Its results show that the direct effect on HRQoL of the medical variable (FEV1) is smaller than the indirect effect, mediated by self-efficacy, optimism and functional impairment (**Fig. 2**).

**Fig. 3:**
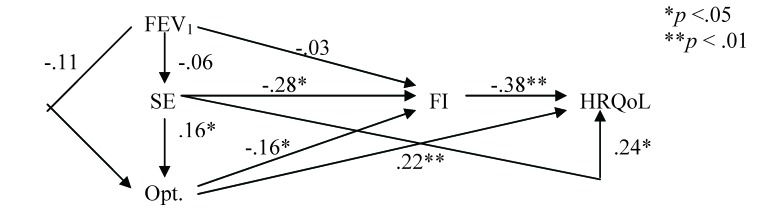
The “reduced" model (the direct effects from FEV1 to QWB were removed). FEV1 = forced expiratory volume in 1s; FI = functional impairment; HRQoL = health-related quality of life; SE = self-efficacy; Opt. = optimism.

 We applied the same procedure within a “reduced" model (**Fig. 3**), where all direct correlations between FEV1 and HRQoL were removed. It showed correlation coefficients of self-efficacy, functional impairment and optimism to HRQoL that were similar to those in the previous “full" model (the indirect correlations were, just as in the “full" model, smaller than direct correlations). 

 In order to test to what extent the “reduced" model is satisfactory, the matrix of correlations in the “full" model correlation matrix was compared to the correlation matrix stemmed from the “reduced" model. The differences between models were not significant (χ2 = 0.067, ns). This is indicative for the possibility to take into account, when assessing the HRQoL in COPD, the psychosocial variables as good or even better predictors than medical variables.

## Discussion

These results indicate that the association between pulmonary function and quality of life was mediated both by self-efficacy and optimism. The positive correlations between self-efficacy, optimism and HRQoL scores, higher than those between FEV1 and HRQoL, suggest that patients with low self-efficacy and optimism may experience greater discomfort, compared to those with high self-efficacy and optimism, at the same decrease of pulmonary function.

 The functional impairment (FI) scores were moderately correlated both with self-efficacy and with HRQoL scores. This shows that, beside FEV1 and self-efficacy, HRQoL in COPD patients may be influenced also by the perceived functional impairment (the stronger patient’s feelings that they can manage functional abilities, the better their perceived HRQoL). However, the direction of causality could be further discussed, as the design of this study was transversal.

 Our results are consistent to the mediational role of psychosocial factors on the relationship between objective symptoms and health-related quality of life, role indicated by Bandura [**8**] as being extremely important, especially in chronic diseases. According to this model, self-efficacy and optimism can lead to (a stronger belief in the efficacy of) various coping strategies, that, in turn, decrease perceived functional disability and increase perceived health-related quality of life. These results are consistent also with previous research focused on COPD and using the same methodology that concluded on the existence of a solid relationship between constructs such as self-efficacy and quality of life in these patients [**16**], and on the importance of self-efficacy expectations and optimism on the 5-year survival [**17**].

### Clinical implications

*Purpose of psychological assessment*

 The information obtained from self-efficacy and optimism scales could be helpful in explaining patient’s behavior in certain instances. This information could also contribute in planning individualized treatment and allow physicians and behavioral scientists to assess the confidence of COPD patients in their ability to avoid or to manage breathing difficulties they may face. Patients’ confidence that they can transform their knowledge into effective action to avoid breathing difficulty seems to be a parameter that is worthy to be invested in, given its significant impact on HRQoL. Furthermore, literature data reveal that interventional strategies, designed to improve self-efficacy in COPD patients have proven to be effective in making patients adopt more healthy behaviors (e.g. avoid smoking) and increase adherence to treatment [**18**].

Interventional strategies

 Self-efficacy and optimism in COPD may be increased via strategies such as systematic desensitization, addressing fears and apprehensions of the patient, and self-management training, which may include supervised training exercises involving the successful utilization of self-efficacy behaviors for the avoidance of smoking and a more constructive approach of breathing difficulties. In selected cases (e.g. onset of frank psychological symptoms), these methods can be complemented by cognitive-behavioral therapy.

 Both biomedical and psychosocial strategies must be taken into account, in order to provide an optimal assessment and a wider, more efficient and personalized treatment in COPD.

**Sources of funding**

 This research was funded from personal sources. 

**Disclosures**

 None.
